# Designing for Improved Patient Experiences in Home Dialysis: Usability and User Experience Findings From User-Based Evaluation Study With Patients With Chronic Conditions

**DOI:** 10.2196/53691

**Published:** 2024-05-14

**Authors:** Anna Aspelund, Paula Valkonen, Johanna Viitanen, Virpi Rauta

**Affiliations:** 1 Department of Computer Science Aalto University Espoo Finland; 2 Department of Nephrology Helsinki University Central Hospital University of Helsinki Helsinki Finland; 3 Abdominal Center Nephrology Helsinki University Hospital Helsinki Finland

**Keywords:** usability, UX, user experience, PX, patient experience, user-based evaluation, patients, eHealth, digital health solution, kidney disease, home dialysis

## Abstract

**Background:**

Chronic kidney disease affects 10% of the population worldwide, and the number of patients receiving treatment for end-stage kidney disease is forecasted to increase. Therefore, there is a pressing need for innovative digital solutions that increase the efficiency of care and improve patients’ quality of life. The aim of the eHealth in Home Dialysis project is to create a novel eHealth solution, called eC4Me, to facilitate predialysis and home dialysis care for patients with chronic kidney disease.

**Objective:**

Our study aimed to evaluate the usability, user experience (UX), and patient experience (PX) of the first version of the eC4Me solution.

**Methods:**

We used a user-based evaluation approach involving usability testing, questionnaire, and interview methods. The test sessions were conducted remotely with 10 patients with chronic kidney disease, 5 of whom had used the solution in their home environment before the tests, while the rest were using it for the first time. Thematic analysis was used to analyze user test and questionnaire data, and descriptive statistics were calculated for the UMUX (Usability Metric for User Experience) scores.

**Results:**

Most usability problems were related to navigation, the use of terminology, and the presentation of health-related data. Despite usability challenges, UMUX ratings of the solution were positive overall. The results showed noteworthy variation in the expected benefits and perceived effort of using the solution. From a PX perspective, it is important that the solution supports patients’ own health-related goals and fits with the needs of their everyday lives with the disease.

**Conclusions:**

A user-based evaluation is a useful and necessary part of the eHealth solution development process. Our study findings can be used to improve the usability and UX of the evaluated eC4Me solution. Patients should be actively involved in the solution development process when specifying what information is relevant for them. Traditional usability tests complemented with questionnaire and interview methods can serve as a meaningful methodological approach for gaining insight not only into usability but also into UX- and PX-related aspects of digital health solutions.

## Introduction

Chronic kidney disease is a global health problem that leads to kidney failure, cardiovascular disease, and premature death. Chronic kidney disease affects 10% of the population and is one of the leading causes of mortality worldwide [1]. Dialysis, along with kidney transplant, is a lifesaving treatment for people with end-stage kidney disease. Dialysis can be delivered in hospital or home settings, and home dialysis is associated with a higher or equal quality of life for patients [2] and lower costs for the health care system [3]. As the number of patients receiving treatment for end-stage kidney disease is forecasted to rise [4], innovative digital solutions that maximize efficiency, improve patients’ quality of life, and facilitate care delivery and monitoring are needed.

eHealth solutions, such as digital patient engagement platforms (DPEPs), are increasingly developed to support self-care, enhance patient-clinician collaboration, and increase the efficiency of care delivery [5-7]. In dialysis care, new DPEP solutions have the potential to improve disease management, health outcomes, and patient experience (PX) among patients with chronic conditions [8,9]. To achieve these goals, a human-centered design approach to development is a necessity. Human-centered design is an approach that aims to make digital systems usable and useful by applying human factors and usability techniques, such as user-based testing, guidelines for interaction design, prototypes, user observations, and user requirements specifications [10]. Usability refers to the interaction between the end user and the system, whereas the user experience (UX) includes aspects like emotions, beliefs, and perceptions [10,11]. Originating from the UX, the PX has also become an important and acknowledged concept as the health care sector has shifted to a more customer-oriented approach. PX has been used to describe patients’ interactions and care experiences across the care continuum [12,13], but it lacks a consensus definition [14]. Regarding eHealth solutions, numerous factors influence PX, such as the solution type and quality, risks and concerns, communication, remote interaction, and patients’ attitudes toward digital solutions [14].

Several studies have evaluated the usability of eHealth solutions aimed at patients with chronic and serious conditions. These have included solutions targeted to patients with cancer for monitoring and managing their illness or treatment-related symptoms [15-17], digital self-management programs for patients with juvenile idiopathic arthritis [18] and chronic obstructive pulmonary disease [19], and an electronic patient-reported outcome tool for patients with complex chronic disease and disability to set and monitor their health-related goals [20]. Common usability problems identified across these studies have included terminology issues [15,18], navigation problems [15,17], and challenges with the way information is presented to the patients [16,18]. Regarding UX, studies have found that patients’ illness-related problems and limitations should be taken into account when designing eHealth solutions for patients with chronic and serious conditions [16,19,20]. Further, customization of the solutions, for example, based on the stage or severity of the illness or type of treatment should be possible to provide a pleasant UX [16,17,19]. Some prior studies have also reported PX-related findings, such as patients fearing that the eHealth solutions will replace in-person consultations with clinicians [20], and patients generally welcoming the additional digital communication channel [16,17]. However, these results have not been analyzed or described in relation to PX, and it seems that PX-related aspects were not systematically explored in the evaluation studies.

In this paper, we report a user-based evaluation study of the novel eHealth solution: a DPEP targeted to patients with chronic kidney disease in CKD stages 4-5, for example, to patients undergoing predialysis and patients undergoing home dialysis (both peritoneal dialysis and home hemodialysis). Patients with functioning renal transplants were excluded. This study is part of the larger eHealth in Home Dialysis project [21], which is coordinated by HUS Helsinki University Hospital, Finland. The solution is designed to facilitate advanced home care: enable patients with chronic kidney disease to document their treatment data, monitor their clinical and health data, order dialysis supplies, and report their symptoms as well as enhance patient-provider communication. The objective of our study was to evaluate the first version of the DPEP solution, called eC4Me, and support deployment of the solution and promote end user participation in later phases of the development. The research questions are as follows: (1) What kind of usability problems does the evaluated DPEP solution have? (2) What kind of UXs, expectations, and improvement ideas do patients with chronic kidney disease have about the new DPEP solution? and (3) How can the new DPEP solution support positive PX for patients with chronic conditions?

Our user-based evaluation study aims to widen the scope of usability evaluations of eHealth solutions targeted at patients with chronic and serious conditions to include PX-related aspects alongside usability and UX. Additionally, to our best knowledge, this is the first study to evaluate a DPEP solution specifically targeted to patients with chronic kidney disease.

## Methods

### Study Design

Our study design was based on a formative user-based evaluation approach [22]. The formative evaluation aims to support the improvements of the system, particularly the user interface, as part of an iterative design process [22]. A typical method of formative evaluation is a think-aloud usability test, which includes 4 stages: preparation, introduction, the test itself, and debriefing [22]. Usability testing is stated to be the most fundamental usability method since it provides direct information about how people use the systems and what their exact problems are [22]. In practice, the think-aloud method involves the test participants continuously thinking aloud while performing the predefined test tasks. The researcher’s role is to make observations and continuously prompt the participant to think aloud by asking general questions [22].

The usability assessment methods recommended for gathering supplementary data are observations, questionnaires, and interviews [22]. For the questionnaire, we used the UMUX (Usability Metric for User Experience) [23], which closely conforms to the 3 widely acknowledged attributes of usability—effectiveness, efficiency, and satisfaction [10]—and strongly correlates with other commonly used usability metrics such as the System Usability Scale [24,25]. UMUX questionnaire is considered compact since it includes four question items: (1) the system’s capabilities meet my requirements, (2) using the system is a frustrating experience, (3) the system is easy to use, and (4) I have to spend too much time correcting things with this system [23].

Due to the COVID-19 pandemic, we conducted the evaluation sessions remotely. Experiences from previous studies have shown that high-quality research data can be collected remotely [26]. However, compared to traditional face-to-face usability testing, remote evaluations require more pedantic preparation. Researchers must pay attention to building trust and confidentiality [26], choose tools that are familiar and easily accessible for the participants [27], focus on body language and facial expressions to establish rapport [26,28], and provide the participants with technical support as needed [29]. Other recommendations include having multiple researchers participate in remote test sessions [28], and using a synchronous approach, which makes it possible to observe participants’ screens in real-time [30].

### Evaluated DPEP Solution

**Figure 1:**
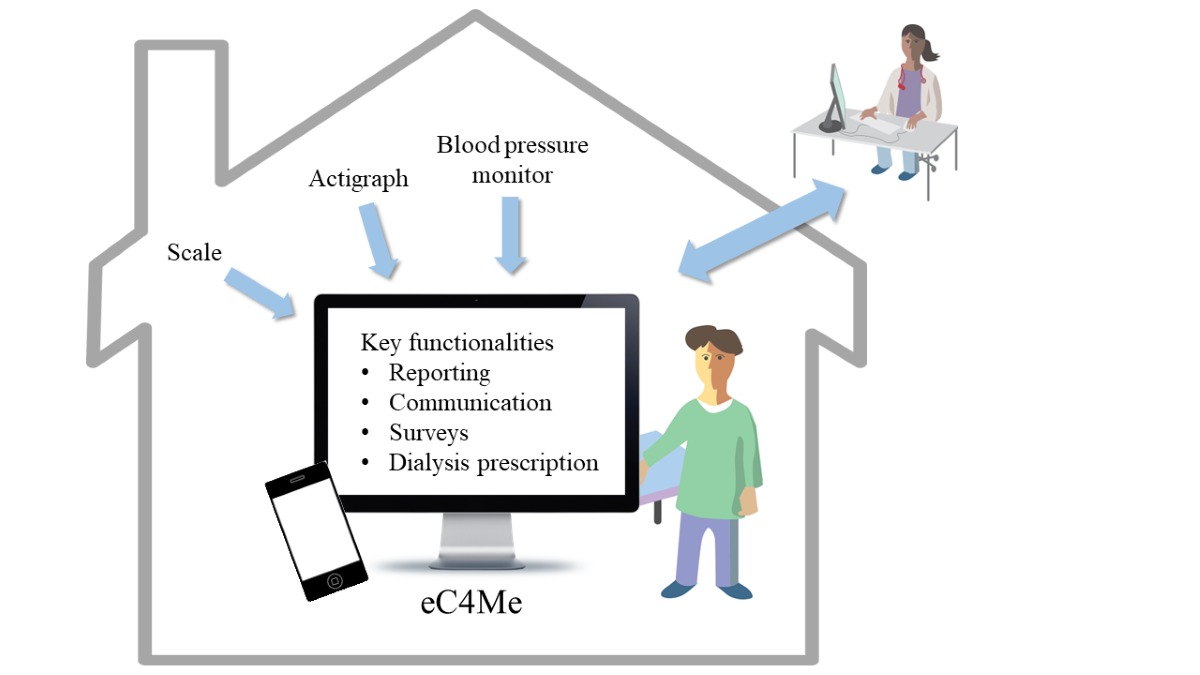
First version of the eC4Me solution.

The first version of the eC4Me solution was introduced in autumn 2021. A core part of the solution was an app, which had both computer and mobile interfaces and included the following key functionalities: monitoring of treatment-related data (reporting functionality), messaging between patients and nurses (communication functionality), answering quality-of-life surveys (survey functionality), and access to patient’s dialysis prescriptions (dialysis prescription functionality; see Figure 1). In addition, the solution delivered to the patients included external monitoring devices, such as a blood pressure monitor, a scale, and an actigraph, which, together with clinical data collected from electronic health records, enabled patients to monitor their conditions.

### Participants

The participants were recruited from a large university-affiliated nephrology clinic with the help of research nurses. All participants were familiar with the eHealth in Home Dialysis project since they had participated in an interview study that was conducted earlier as part of the project.

Eleven patients with chronic kidney disease were originally invited to participate in this study. As 1 test participant was particularly interested in technology development and came to his test session with predrafted design ideas, we decided to modify his test session to focus on discussing these ideas. Consequently, he did not perform the test tasks in our test procedure, and the data from his session was omitted from this study. Therefore, data from 10 user-based evaluation sessions were included in this study.

The background information collected from the participants included age, gender, type of treatment, occupational status, and technical skills. Technical skills were evaluated by asking participants to give their own estimation of their technical skills, with response options being “good,” “basic,” and “weak.” Half of the participants (n=5) had the solution delivered to their homes 1-3 weeks before the tests, whereas the other half (n=5) were using the solution for the first time in their test session. Participant characteristics are shown in Table 1.

**Table 1:** Participant characteristics (N=10).

Characteristics			Participants, n (%)
**Type of treatment**			
	Predialysis (not yet in dialysis treatment)	4 (40)	
	Peritoneal home dialysis	3 (30)	
	Home hemodialysis	3 (30)	
**Experience with the solution before the test session**			
	No experience	5 (50)	
	1-3 weeks of experience	5 (50)	
**Sex**			
	Male	6 (60)	
	Female	4 (40)	
**Age (y)**			
	30-60	4 (40)	
	>60	6 (60)	
**Occupational status**			
	Fully working	2 (20)	
	Partially working	2 (20)	
	Not working (fully retired or sick leave)	6 (60)	
**Technical skills (own estimation)**			
	Good	2 (20)	
	Basic	7 (70)	
	Weak	1 (10)	

### Test Procedure

In our study, we used synchronous remote usability testing with Microsoft Teams as a tool, and 2 researchers were present in each session. During the test sessions, patients used the eC4Me solution with a computer, enabling the researchers to monitor their task performance via screen share on Microsoft Teams.

Each test session followed the traditional structure and stages of usability testing [22], lasted about 2 hours, and included the following phases: (1) introduction—participants were introduced to the evaluation study and given an opportunity to become familiar with Microsoft Teams; (2) test tasks—run-through of predefined usability test tasks, which included logging in, searching for information and functionalities, viewing and interpreting health-related data, reporting treatment-related data, and filling in the surveys; (3) questionnaire—participants answered the UMUX questionnaire; and (4) interview—participants answered semistructured interview questions to elaborate their UMUX scores and give overall feedback on the solution based on the usability test tasks. The interview consisted of 4 open-ended “Why did you give this score?” questions, which were asked for each of the UMUX items separately, and a question on how participants would improve the solution.

Before the actual tests, the test procedure was piloted with 2 research nurses. To ensure privacy, all patients used the solution with test login IDs and dummy health data during the test sessions. The exact test tasks varied slightly between the participants, depending on their prior experience with the solution, illness stage, and type of treatment, as not all functionalities of the solution were relevant for all patients. The participants who had used the solution before the tests were also encouraged to provide feedback on the entire solution including the research devices and a mobile interface. This study was performed in the Finnish language.

### Data Analysis

The qualitative data included video recordings from remote usability tests, observation notes, and transcripts from semistructured interviews. The qualitative data were analyzed following a thematic analysis method [31], which involved collaboration between 3 researchers (AA, PV, and JV). The data were first coded by 1 researcher (PV), and the findings were discussed by the 3 researchers. Further, 2 researchers (AA and JV) then continued the analysis by categorizing the codes into thematic groups, following the principles of the affinity diagram method [32]. The following main three thematic groups were used: (1) *usability*, which includes findings about users’ interactions with the DPEP solution; (2) *UX*, which includes findings about users’ experiences and feelings toward using the DPEP solution; and (3) *PX*, which includes findings about how the DPEP solution can support patients’ interactions and care experiences across the care continuum.

The researchers (AA and JV) then continued the analysis with several rounds of iterations. Along with other data, improvement ideas expressed by the participants were thematically grouped. At the end of the analysis, the thematic grouping of observations was discussed, approved, and finalized collaboratively by the 3 researchers.

The quantitative data consisted of UMUX item scores, which were analyzed following the UMUX scoring scheme [23]: to obtain the overall UMUX score, items 1 and 3 were scored as [score−1] and items 2 and 4 as [7−score], and the sum of the item scores was then divided by 24 and multiplied by 100. In addition to the overall score, the means and SDs for each of 4 question items were calculated separately for 2 participant groups (patients who had or had not used the solution before the test). The differences between the groups were analyzed using *t* tests for independent samples. The tools used for data analysis were ATLAS.ti (ATLAS.ti Scientific Software Development GmbH) and Microsoft Excel for qualitative data analysis and Microsoft Excel for statistical analysis.

### Ethical Considerations

This study has a research permit from the ethical committee of the Hospital District of Helsinki and Uusimaa (HUS/1649/2020).

## Results

### Overview

The results are divided into 5 topics: usability, UX and PX findings each, UMUX results, and improvement ideas.

### Usability

The *usability* findings of the evaluated eC4Me solution consisted of 8 subthemes (Table 2).

*Navigation* includes findings about whether patients could locate the functionalities, content, and commands that they were looking for. Nine out of 10 users had at least some problems navigating the app, and the most common navigation challenges were related to users not understanding the content structure or the terminology used in the menus.

**Table 2:** *Usability*, *UX*, and *PX* findings of the user-based evaluation. “All findings” includes positive, negative, and neutral findings. For the *usability* theme, negative findings, that is, the identified usability problems, are also reported separately under “problems.”

Subtheme			All findings				Problems		
			Codes, n		Users, n		Codes, n		Users, n
**Usability**									
	Navigation	41		10		19		9	
	Terminology	35		10		22		8	
	Front page	21		10		3		2	
	Presentation of data	35		9		17		5	
	Login	10		9		4		4	
	Survey functionality^a^	12		7		2		2	
	Reporting functionality^a^	50		6		29		6	
	Dialysis prescription functionality^a^	6		5		0		0	
**UX^b^**									
	Technical functionality	26		9		—^c^		—	
	Use of access devices (computer, tablet, or mobile)	26		9		—		—	
	Workload and effort	16		7		—		—	
	Perceived benefits	12		7		—		—	
	Security	8		4		—		—	
**PX^d^**									
	Content-related needs	67		10		—		—	
	Situation of use	57		10		—		—	
	Communication with clinicians	52		10		—		—	

^a^The survey, reporting, and dialysis prescription functionalities were tested with some of the participants only (n=7, n=6, and n=5, respectively).

^b^UX: user experience

^c^Not applicable.

^d^PX: patient experience

*Terminology* includes findings about the comprehensibility and clarity of the terminology used. Eight users (80%) had problems understanding the terminology, and approximately half (10/22) of the terminology challenges were related to problems with understanding medical- or treatment-related terminology. Other terminology issues included problems with the terms used in the menus as well as the use of a foreign language.

*Front page* includes findings about the comprehensibility and clarity of the front-page contents. The front page of the tested version contained relatively little information and functionalities, and most users found it simple and clear.

*Presentation of data* includes findings about the comprehensibility and clarity of the presentation of health data, such as health measurements. Five users (50%) had issues understanding or viewing the data. The most common challenges were not comprehending the data or graphs or not knowing how to adjust the scales and timelines to view the data in a meaningful way.

*Login* includes findings about the ease of logging in. Four users (40%) had problems logging into the system. Typical challenges included not understanding where to input the login information or making errors while typing the login details.

*Surveys, reporting, and dialysis prescription functionalities* include findings about the ease of use of these functionalities. All users who tested the reporting functionality (6/6) had problems using it. Users struggled with not understanding what they should type in the input fields, feeling that options in the fields did not match the way treatment was provided in the real world, or not comprehending the medical- or treatment-related terminology. In this study, there were few usability issues in the survey functionality and none in the dialysis prescription functionality.

### About UX

The *UX* findings of the evaluated eC4Me solution consisted of 5 subthemes (Table 2).

*Technical functionality* includes patients’ experiences and feedback regarding the technical aspects of the eC4Me solution. Four users (40%) expressed frustration because some information they thought should be transferred automatically between the app, the research devices, the home dialysis machine, and patient information systems had to be typed manually. For the same reason, 2 users (20%) felt that they needed to use several systems for essentially the same purpose, such as monitoring their health data.

*Use of access* devices includes patients’ expressed preferences regarding using the solution with different access devices: desktop computer, tablet, or mobile phone. Two users (20%) said they would prefer to use the computer interface, as they have found tablet and mobile keyboards difficult to use or feel that the mobile interface would give them less information. In contrast, 3 (30%) users indicated that they preferred a mobile phone or tablet as they are readily at hand and easier to use during the treatment, while another 3 (30%) said that their choice of access device would depend on the task they were performing.

*Workload and effort* includes findings about the perceived time and effort required to use the solution. Six users (60%) felt that the solution was not burdensome to use as such and that filling in the surveys or documenting treatment details did not take too much time. However, 4 users (40%) expressed concern that the solution might nevertheless increase their burden if it does not replace any other service, thus becoming one more thing to use and keep track of on top of all the other health-related solutions.

*Perceived benefits* includes patients’ thoughts about the benefits and added value of the eHealth solution. One (10%) user saw value in using the solution primarily for the benefit of the health care personnel, while 2 (20%) others said that they needed to see clear benefits for themselves to be motivated to use the solution. Yet another user mentioned that the data generated by the solution could benefit all patients, as it could be used for research and treatment development.

I’m uncertain what this is meant for, is it for my benefit or someone else's? The remote measuring devices that I have had, I have found the data very useful for myself.... But I don’t understand the thinking behind this (the solution), do I benefit or is it someone else?

P7

*Security* includes findings about potential security issues and patients’ concerns regarding the use of the eHealth solution. Only 2 (20%) users gave direct comments on security aspects, while most findings related to security were observations of behaviors that could introduce potential security risks, such as the user closing the browser instead of logging out when asked to do so.

### About PX

The *PX* findings of the evaluated eC4Me solution consisted of 3 subthemes (Table 2).

*Content-related needs* includes patients’ comments regarding health-related data that they want to see so they can monitor and manage their treatment and health. The expressed needs and what was considered most important varied between the users, but overall, patients were interested in seeing all the types of data that the tested version of the solution provided. Only 1 user (10%) gave a general comment that the solution “should not contain anything unnecessary or useless,” but other than that, none of the users reported that they would not need or want to see some of the information or data that was available to them.

*Situation of use* includes patients’ comments and feedback about how well the solution fits their situations and supports their everyday lives with the disease. Users had numerous, often variable comments regarding how often and in what situations they would likely use the solution. They also commented on how well the functionalities fit their care and treatment schedules, as in the following quote:

I fill these during my home dialysis treatment, so I may write notes about yesterday’s treatment. I don’t necessarily have time to use [the solution] after the treatment.

P8

*Communication with clinicians* includes findings about how the new solution supports patient-clinician communication. Users expressed interest in using the messaging function and saw benefits in using the documented data to facilitate their communication with clinicians during face-to-face appointments. It was not clear to the users how actively and by whom their data were being monitored and if messages were noticed and replied to. Three users (30%) were hoping for immediate feedback, while 4 others (40%) considered the messaging function appropriate for nonurgent communication. In addition, 5 users (50%) expected their own nurse to read and respond to their messages, while 3 users (30%) thought that the work was handled by a care team.

### UMUX Results

The UMUX score of the first version of eC4Me was 70.6 (SD 18.6), which indicates an average level of usability [25].

Means for individual UMUX items are presented in Table 3. Users with 1-3 weeks of prior experience with the solution rated it more favorable overall compared to users without prior experience. However, the differences between the groups were not statistically significant.

**Table 3:** UMUX^a^ item scores per user groups on a scale of 1 “strongly disagree” to 7 “strongly agree.”

UMUX questionnaire item	All users (n=10), mean (SD) score	Users with no prior experience using the solution (n=5), mean (SD) score	Users with 1-3 weeks of experience using the solution (n=5), mean (SD) score
The solution’s capabilities meet my requirements	4.5 (1.4)	4.0 (1.6)	5.0 (1.0)
Using the solution is a frustrating experience	2.5 (1.9)	3.0 (2.3)	1.9 (3.0)
The solution is easy to use	5.7 (1.5)	5.0 (1.9)	6.4 (0.5)
I have to spend too much time correcting things with the solution	2.8 (1.9)	3.0 (1.4)	2.6 (2.5)

^a^UMUX: Usability Metric for User Experience.

### Improvement Ideas

In total, 66 improvement ideas (Table 4) for the eC4Me solution were identified from the data, with all 10 users expressing at least one improvement idea. Two-thirds of the ideas (40/66) came from users who had used the solution before the test.

The most common theme for improvement ideas was *content-related needs*. Seven patients (70%) expressed interest in monitoring some health-related measurements that were not available in the tested version, and 3 (30%) patients wanted to see benchmark values or descriptions that would enable them to better understand their health data.

**Table 4:** Improvement ideas and their most common subthemes.

Subtheme	All improvement ideas	
	Codes, n	Users, n
Content-related needs	28	9
Situation of use	15	7
Communication with clinicians	15	6
Presentation of data	8	6
Ease of using reporting	13	5
Technical functionality	8	5
Other^a^	26	8

^a^Subthemes with fewer than 5 ideas (combined).

In addition, the participants brought up improvement ideas related to the following: (1) *situation of use—*ideas on how the solution could be improved to better fit the patient’s situation, everyday life, and treatment schedule; (2) *communication with clinicians—*ideas on how the solution could better support communication and data exchange between patients and clinicians; (3) *presentation of data*—ideas on how health data could be presented to make them more meaningful for the patients; (4) *ease of using reporting*—ideas on how to improve the reporting functionality to make it easier to use; and (5) *technical functionality*—ideas regarding automatic data exchange between the solution and other devices or services.

## Discussion

### Main Contribution

Our user-based evaluation study of the novel DPEP solution targeted to patients with chronic kidney disease with 10 participants resulted in a wide variety of usability-, UX-, and PX-related findings.

Most usability problems of the first version of the solution were related to navigation, the use of terminology, and the presentation of health data. Many participants struggled with the reporting functionality, which was one of the key functionalities of the solution. A considerable number of patient participants also expressed improvement ideas related to these themes. We decided not to classify usability problems by severity, as a proper severity rating should consider not only usability aspects but also potential medical- and health-related consequences of users’ mistakes and misunderstandings. However, the usability challenges identified in our study were remarkably similar to those found in evaluations of other eHealth solutions aimed at patients with chronic and serious conditions [15-18]. Our findings thus emphasize the importance of using terminology and presenting health data in a way that is understandable and meaningful to patients. Our results also highlight the need to consider patients as end users when designing user interfaces for eHealth solutions.

Our study identified several challenges related to the UX of the evaluated DPEP solution. Largely due to deficiencies in integration and data exchange, the participants feared that the solution might create additional tasks and thus increase their burden. Our results also showed considerable variation in the expected benefits of the solution. Some patients wanted to see direct value for themselves, whereas others mentioned benefits for the health care professionals as their primary motivation of use.

Despite the usability and UX challenges, the patients’ overall ratings of the evaluated solution were surprisingly positive. This may be at least partially explained by findings from previous studies, which have shown that patients with chronic and serious conditions often express high interest in disease-specific eHealth solutions [17,18], even when experiencing severe usability challenges [20]. In our study, patients who had used the solution for a few weeks in a home setting evaluated it more positively than patients who were using the solution for the first time. Although the differences were not statistically significant due to the small number of participants, these initial findings could simply be explained by the fact that learning to use the solution makes it easier and thus more pleasant to use. However, they could also indicate that after having used the solution in their home setting with their own health data, patients have a better understanding of the benefits and potential value of the solution.

Regarding PX, our study generated insights on how the DPEP solution can support patients with chronic conditions in monitoring and managing their conditions and how the DPEP solution could better fit their everyday lives with a disease. From the patients’ perspective, it is not enough that an eHealth solution is easy or pleasant to use if it does not support their health-related goals, feel meaningful, and fit their real-life situations and daily care activities. Special attention needs to be paid to ensure that these PX-related considerations are included in user-based evaluations of eHealth solutions, as generic usability questionnaires, classifications, and frameworks do not adequately capture these aspects [33,34]. As our study shows, traditional usability tests, complemented with questionnaire and related interview methods, can serve as a meaningful methodological approach for collecting information about PX-related aspects of eHealth solutions.

In our study, the participants generated a considerable amount of improvement ideas. In particular, nearly all patients had ideas on what health-related data they would like to see to better manage their condition. This implies 2 things. First, many patients with chronic conditions are interested in taking responsibility for their own care. Although the participants selected for our study are likely to represent the most motivated and active patients with chronic kidney disease, it would seem meaningful to support and empower these motivated patients to take more responsibility by providing them with the information they view as important and meaningful, not only the information that makes the most sense from a health care professionals’ point of view. Second, patients should be actively involved in the co-design process in the early phases of solution development and when specifying what kind of information is relevant for them.

The user-based evaluation was a crucial step in the eHealth solution development process and generated findings that helped to make substantial changes in the solution to make it more suitable for the end users (patients), thus helping the solution reach its goals. The evaluation of usability-, UX-, and PX-related aspects of the solution will continue in a future research project. We aim to conduct a similar study in a further phase of the development project to examine how the usability and UX of the solution have been improved.

### Limitations

Due to the COVID-19 pandemic, our study used remote testing as an evaluation method. In comparison to in-context evaluations, this limited the scope of our evaluation, as we could not fully observe participants in their home environments. We also decided not to include research devices that were part of the DPEP solution in the test procedure, as this would have been difficult to realize in the remote setup. However, when compared to face-to-face usability testing in laboratory settings, our arrangement also had some advantages. As contextual factors are well known to influence emotional experiences and expressions [35], allowing patients to remain in their natural home settings during test sessions likely produced more reliable data, especially regarding experience-related topics like UX and PX.

### Further Research

Our findings indicate that including participants who have used the evaluated solution before the test can have a nonnegligible effect on the quality and amount of information that the evaluation study generates. Half of the participants in our study had used the evaluated solution in their home environment with their own health data, while the other half were using the solution for the first time in their test session. As the number of participants was small (n=10) and the groups were heterogeneous in terms of other background variables, it was not meaningful to make more comprehensive comparisons between the 2 groups. However, participants with prior experience evaluated the solution more favorably and generated more improvement ideas. Many UX- and PX-related aspects, such as perceived benefits, workload, and compatibility with everyday life, can be difficult to assess using a solution only in a test setting, especially considering that privacy issues often prevent researchers from using patients’ own health data in user tests. This could have implications on how user-based evaluation studies of eHealth solutions should ideally be arranged, and it is therefore an important topic for further research.

In addition, further research is needed to explore the relationship and connections between the concepts of UX and PX, as suggested by recent review studies [14,36]. This includes planning and practicalities of user-based evaluation studies, considering the PX perspective, for the assessment and improvement of eHealth services.

### Conclusions

User-based evaluation can produce valuable findings about usability aspects but also about the UX and PX of the evaluated DPEP solution. The findings of our study can be used in the development process to improve the evaluated solution from the perspective of patients with chronic conditions. Evaluation is a useful and necessary part of the solution development process, especially considering the high number of novel eHealth solutions that are currently being developed.

Our study also highlights the importance of understanding how digital health solutions for patients with chronic and serious conditions support patients’ own health-related goals and fit their lives with disease. To fully understand the motivation for using such solutions, it is necessary to understand how patients perceive the benefits versus the effort required to use the solution in their everyday lives.

## Abbreviations

DPEP: digital patient engagement platform
PX: patient experience
UMUX: Usability Metric for User Experience
UX: user experience
